# Posterior Cord Syndrome and Trace Elements Deficiency as an Uncommon Presentation of Common Variable Immunodeficiency

**DOI:** 10.1155/2017/9870305

**Published:** 2017-03-05

**Authors:** Yuri Silva Macedo, Ananda dos Santos Mota, Priscila Morais Monteiro, Angela Cristina Gouvêa Carvalho, Barbara Fernandes Diniz, Pedro Gemal Lanzieri, Ricardo Carneiro Ramos, Luis Otavio Mocarzel, Ronaldo Altenburg Gismondi

**Affiliations:** Department of Clinical Medicine, Hospital Universitário Antônio Pedro (HUAP), Universidade Federal Fluminense (UFF), Rua Marquês de Paraná 303, 7° Andar, Centro, 24033-90 Niterói, RJ, Brazil

## Abstract

Diarrhea is one of the most common symptoms in common variable immunodeficiency, but neurologic manifestations are rare. We presented a 50-year-old woman with recurrent diarrhea and severe weight loss that developed a posterior cord syndrome. Endoscopy found a duodenal villous blunting, intraepithelial lymphocytosis, and lack of plasma cells and magnetic resonance imaging of the spine was normal. Laboratory assays confirmed common variable immunodeficiency syndrome and showed low levels of trace elements (copper and zinc). Treatment was initiated with parenteral replacement of trace elements and intravenous human immunoglobulin and the patient improved clinically. In conclusion, physicians must be aware that gastrointestinal and neurologic disorders may be related to each other and remember to request trace elements laboratory assessment.

## 1. Introduction

Common variable immunodeficiency (CVID) is the most common primary immunodeficiency in clinical practice, with an incidence of 1/10,000 to 1/50,000 [1, 2]. CVID comprises a heterogeneous group of diseases characterized by abnormal antibody production and [2, 3] decreased production of IgG, IgA, and/or IgM, as well as impaired antibody response to both polysaccharide and protein antigens [1]. As a result of hypogammaglobulinemia, most CVID patients have recurrent infections, autoimmune diseases, lymphoproliferative, granulomatous or neoplastic disorders, and intestinal dysfunctions [1]. Over 90% of the patients present with recurrent bacterial infections, mainly of the respiratory and gastrointestinal tracts.

Diarrhea is one the most common symptoms in CVID and can be caused by a myriad of diseases. Recurrent infections and intestinal wall inflammatory dysfunction are two usual etiologies of diarrhea. On the other hand, neurological manifestations in CVID are rare. Among neurologic diseases, meningitis due to encapsulated bacteria is relatively common. However, transverse myelitis, peroneal muscular atrophy, Guillain-Barré syndrome, and myasthenia gravis were also reported.

Gastrointestinal and neurologic disorders may be related to each other. Cobalamin deficiency may occur because of intestinal malabsorption, causing anemia and neurologic disorders such as cognitive dysfunction, posterior spinal cord syndrome, and/or peripheral neuropathy. In addition, malabsorption of other vitamins and trace elements may also cause neurologic disease. However, among previous reports of trace elements deficiency in CVID none manifested as neurologic disease. We report the case of a 50-year-old woman with CVID and chronic diarrhea whose neurologic manifestations were due to intestinal malabsorption of trace elements.

## 2. Case Presentation

A 50-year-old Brazilian white woman presented to an outpatient clinic with a history of frequent episodes of diarrhea over the last three years, with presence of food debris, postprandial fullness, and significant weight loss. Patient denied fever, blood, or mucus in stool. Physical examination was normal. Patient underwent stool examination and upper and lower gastrointestinal (GI) endoscopy that were normal. In addition, antiendomysial gliadin and transglutaminase antibodies were also negative. Treatment with probiotics and a gluten-free diet was ineffective.

**Figure 1:**
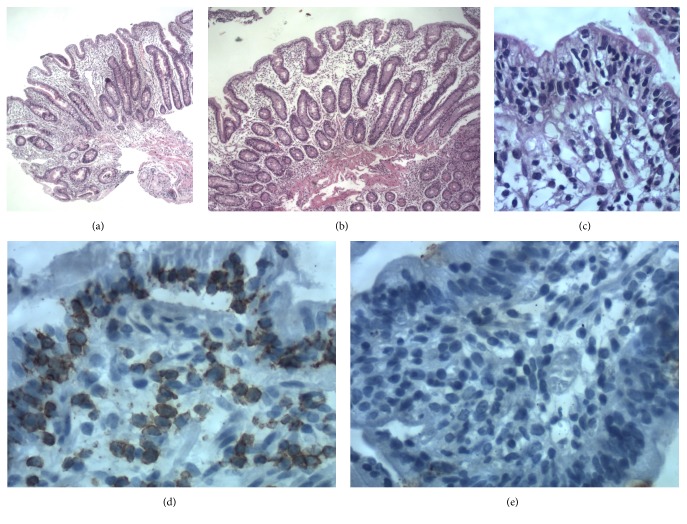
(a) Duodenal villous blunting, HE stain, 10x. (b) Duodenal villous blunting, oedema, HE stain, 10x. (c) Intraepithelial lymphocytosis and lack of plasma cells, HE stain, 10x. (d) Immunohistochemistry CD3-T lymphocytes positive. (e) Immunohistochemistry MUM 1 plasma cells negative.

**Figure 2:**
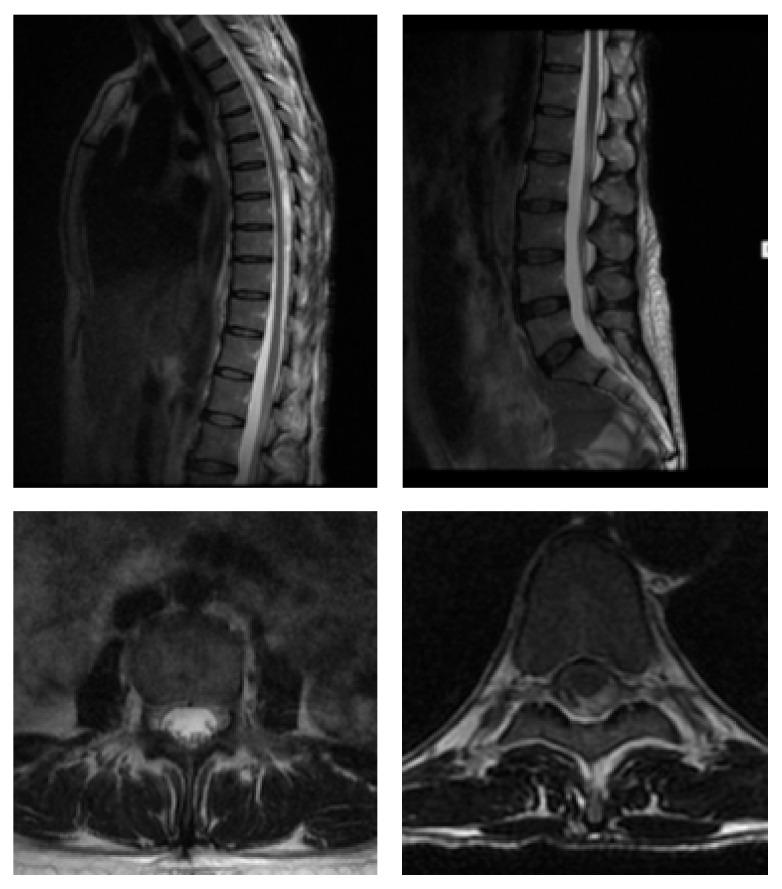
Sagittal and axial T2 weighted MRI images. Vertebral height, disc height, alignment, and bone marrow signal are within normal limits. The canal and neural exit foramina are capacious at all levels.

Over the next months she developed asthenia, paraesthesia, and infrapatellar edema in the lower limbs. A new neurologic examination showed positive Romberg sign, ataxic gait, and loss of balance and patient was admitted to our institution. Admission laboratorial exams demonstrated anemia and electrolyte imbalance (Table 1) and low levels of serum immunoglobulins (Table 2). ECG showed sinusal rhythm and a first-degree atrioventricular block. We repeated GI endoscopies: upper GI endoscopy found a chronic duodenitis with villous atrophy and intraepithelial lymphocytosis and lack of plasma cells (Figure 1). No evidence of deposits was found, ruling out amyloidosis. Colonoscopy demonstrated ileitis with eosinophilia and nodular lymphoid hyperplasia and colon and rectal biopsies were almost normal. No parasites were found. Magnetic resonance imaging of the spine was normal (Figure 2). At this moment, we looked for the etiology of neurologic manifestations and trace elements assay showed low levels of copper and zinc. Treatment was initiated with intravenous human immunoglobulin in monthly doses and daily parenteral replacement of trace elements. The patient improved clinically and was discharged for outpatient follow-up.

## 3. Discussion

We describe the case of a previously healthy patient with chronic diarrhea and significant weight loss associated with symptoms of posterior spinal cord syndrome. CVID comprises a heterogeneous group of diseases characterized by abnormal antibody production. It affects men and women equally and the clinical manifestations may begin at any age [3]. In patients older than 4 years, diagnosis of CVID is based on a significant decrease of IgG associated with a decrease of IgA and/or IgM isotypes, in absence of isohemagglutinins and/or poor response to polysaccharide vaccines. Moreover, it is important to exclude other defined causes of hypogammaglobulinemia [3–5]. In CVID, gastrointestinal disorders, autoimmune manifestations, and cancer are more common than in the general population, usually occurring more than 10 years after diagnosis [4, 5].

Recurrent infections are CVID most common clinical manifestation. These infections usually occur at respiratory and gastrointestinal tract. Beside recurrence, atypical pathogens and more severe course are key points to diagnosis. Bacterial infections by encapsulated organism, such as* Streptococcus pneumoniae* and* Neisseria* sp., as well as* Giardia* infections, are particularly common. In addition to recurrent infections, patients with CVID have evidence of immune dysregulation leading to autoimmune and inflammatory disorders and malignant disease. Patients may suffer from chronic lung disease, gastrointestinal and liver disorders, granulomatous infiltrations of several organs, lymphoid hyperplasia, splenomegaly, or malignancy. Our primary hypothesis for patient's diarrhea was an infectious cause. However, blood and stool examinations were negative for both pathogenic bacteria and parasite, including* Giardia*. This way, we looked for noninfectious causes.

Over 60% of patients with CVID have digestive disorders [6–9], such as atrophic gastritis, gastric achlorhydria, imbalance of the intestinal microbiota, parasitosis, lactase deficiency, and malabsorption of cobalamin [6, 8–10]. The most common gastrointestinal manifestation is recurrent diarrhea, and steatorrhea occurs in up to 20% of patients [9, 10]. The main histological alterations in these patients are villous atrophy, intraepithelial lymphocytosis, and lack of plasma cells [8, 9]. The patients' upper and lower GI endoscopy showed nonspecific alterations that are very common in CVID. One important aspect was the exclusion of celiac disease by specific serology, the absence of plasma cells in the duodenal inflammatory infiltrate, and nonresponse to a gluten-free diet. Serum immunoelectrophoresis confirmed the diagnosis of CVID and appropriate therapy (intravenous immunoglobulin) was prescribed.

An increasing awareness of neurologic problems in common variable immunodeficiency has been reported, despite lack of data about its incidence. In the series of Webster, meningitis was described as the most common neurologic manifestation [6]. Epilepsy, mental retardation, ophthalmic zoster, transverse myelitis, subacute combined degeneration of the cord secondary to cobalamin deficiency, schizoaffective psychosis, and peroneal muscular atrophy (Charcot-Marie-Tooth) were reported less frequently in the same group of patients. In another study, a patient with common variable immunodeficiency developed acute disseminated encephalomyelitis [11].

A high prevalence of autoimmune manifestations characterizes common variable immunodeficiency [11]. Therefore, it is important to differentiate autoimmune neurologic symptoms from other etiologies, such as infection, toxic chemicals, and cobalamin and trace elements deficiency. In our patient, the pattern of neurologic manifestations, spinal cord imaging, and laboratory results were key aspects to exclude an autoimmune etiology for posterior spinal cord syndrome.

Posterior cord syndrome is a condition caused by lesion of the posterior portion of the spinal cord, responsible for proprioceptive sensibility. Main signs and symptoms are loss of proprioception and vibration sensation, ataxic gait, positive Romberg sign, hypotonia, and abolition of deep tendon reflexes. Our patient developed most of these symptoms. Posterior cord syndrome can be caused by systemic diseases, such as syphilis and deficiency of cobalamin and trace elements, such as copper, zinc, and aluminum. In a series of 13 cases of CVID, Agarwal et al. observed reduced serum zinc levels in most patients [12, 13]. In addition, there is a report of a patient with CVID and both copper and zinc deficiency. However, these studies did not relate trace elements deficiency and neurologic disorders. Previous studies in general population suggest that low copper promotes neurologic damage, but low zinc levels are usually related to skin and immune dysfunction [14, 15]. In our patient, serum cobalamin level was normal, but zinc and copper were reduced. Therefore, we prescribed trace elements and patient improved.

Common variable immunodeficiency is a big challenge for doctors in primary care centers. The myriad of clinical symptoms and associated disorders can cause delays in diagnosis and treatment. In this case we highlighted the association of gastrointestinal and neurologic disease due to trace elements deficiency. Studies of trace elements malabsorption in CVID are much desirable. Moreover, physicians must be aware that gastrointestinal and neurologic disorders may be related to each other and remember to request trace elements laboratory assessment.

## Figures and Tables

**Table 1:** Laboratorial exams.

Exams	Day 0	Day 10	Day 14	Day 21
Hb (g/L)	126	94	95	93
HCT (%)	39.9	32.8	33.2	30.7
VCM (fL)	73	74		
Leucocytes (cel/mm^3^)	16,310	8,800	8,400	7,200
Platelets (platelets/mm^3^)	572.000	415.000	367.000	282.000
CRP (mg/dL)	3.59	0.19	3.00	
Glucose (mmol/dL)	4.77	4.22	4.66	4.66
Urea (mmol/dL)	11.78	6.43	2.86	7.50
Creatinine (*µ*mol/L)	7.07	4.42	4.42	5.30
Na (mmol/L)	142	141	141	141
K (mmol/L)	3.0	3.3	4.5	3.7
Mg (mmol/L)		0.4	1.0	0.65
Ca (mmol/L)		2.08		2.15
AST (*µ*kat/L)	0.42	0.73	0.95	
ALT (*µ*kat/L)	0.92	1.39	1.75	
Serum protein (g/L)	58	43	45	43
Albumin (g/L)	37	23	25	23
Cobalamin (pmol/mL)	379.9			
Folic acid (nmol/L)	7.6			
Copper (*µ*mol/L)				3.61
Zinc (*µ*g/dL)				52
Aluminum (*µ*g/L)				2
Iron (*µ*g/dL)		41		
Ferritin (ng/mL)		104		

ALT: alanine aminotransferase; AST: aspartate aminotransferase; Ca: calcium; CRP: c-reactive protein; Hb: hemoglobin; HTO: hematocrit; K: potassium; Mg: magnesium; and Na: sodium.

**Table 2:** Serum immunoglobulins assays.

Parameter	Value
IgA (mg/dL)	<6.6
IgG (mg/dL)	232.0
IgM (mg/dL)	9.5

IgA: immunoglobulin A; IgG: immunoglobulin G; and IgM: immunoglobulin M.

## References

[1] Patuzzo G., Mazzi F., Vella A. (2013). Immunophenotypic analysis of B lymphocytes in patients with common variable immunodeficiency: identification of CD23 as a useful marker in the definition of the disease. *ISRN Immunology*.

[2] Cunha V., Moises C., Naves V., Dionigi P., Menezes M., Forte W. (2012). Good evolution of granulomatous form of the common variable immunodeficiency. *Revista Brasileira de Alergia e Imunopatologia*.

[3] Coraglia A., Galassi N., Fernández Romero D. S. (2016). Common variable immunodeficiency and circulating T_FH_. *Journal of Immunology Research*.

[4] Park J. H., Resnick E. S., Cunningham-Rundles C. (2011). Perspectives on common variable immune deficiency. *Annals of the New York Academy of Sciences*.

[5] Divino P. H., Basilio J. H., Fabbri R. M., Bastos I. P., Forte W. C. (2015). Bronchiectasis caused by common variable immunodeficiency. *Jornal Brasileiro de Pneumologia*.

[6] Webster A. D. B., Webster A. D. B. (2001). Common variable immunodeficiency. *Immunology and Allergy Clinics of North America*.

[7] Cunningham-Rundles C., Bodian C. (1999). Common variable immunodeficiency: clinical and immunological features of 248 patients. *Clinical Immunology*.

[8] Errante P., Condino-Neto A. (2008). Common variable immunodeficiency: a comprehensive review. *Revista Brasileira de Alergia e Imunopatologia*.

[9] Torres J., Fortuna J., Trigo E., Lopes A., Campos M. J., Ferreira M. R. (2007). Diarréia num doente com Imunodeficiência Comum Variável: a propósito de um caso clínico. *Jornal Português de Gastrenterologia*.

[10] Kobata C., Neves M. C., Dutenhefner S. E., Silva F. S., Bianchi P. F., Kanashiro E. H. (2000). Manifestação intestinal rara em paciente com imunodeficiência comum variável e estrongiloidíase. Relato de caso. *Revista de Medicina (São Paulo)*.

[11] Özdemir Ö., Okan M. S., Kilic S. S. (2012). Chronic inflammatory demyelinating polyneuropathy in common variable immunodeficiency. *Pediatric Neurology*.

[12] Agarwal S., Mayer L. (2013). Diagnosis and treatment of gastrointestinal disorders in patients with primary immunodeficiency. *Clinical Gastroenterology and Hepatology*.

[13] Litzman J., Dastych M., Hegar P. (1995). Analysis of zinc, iron and copper serum levels in patients with common variable immunodeficiency.. *Allergologia et Immunopathologia*.

[14] Kumar N. (2016). *Copper Deficiency Myeloneuropathy*.

[15] Abrams S. . UpToDate. 2007 (2007). *Zinc Deficiency and Supplementation in Children and Adolescents*.

